# Mechanism-guided pharmacotherapy for cardiometabolic multimorbidity: from pathophysiology to phenotype-prioritized treatment

**DOI:** 10.3389/fendo.2025.1724965

**Published:** 2025-12-01

**Authors:** Hezeng Dong, Liping Chang, Tenghui Tian, Rui Shi, Keying Yu, Cheng Wang, Zhaozheng Liu, Qu Jin, Jing Wang, Tingyu He, Hao Chen, Xiao Shao, Yue Deng

**Affiliations:** 1 Changchun University of Chinese Medicine, Changchun, Jilin, China; 2 Affiliated Hospital of Changchun University of Chinese Medicine, Heart Disease Center, Changchun, Jilin, China

**Keywords:** type 2 diabetes, cardiometabolic multimorbidity, atherosclerotic cardiovascular disease, mechanisms, treatment optimization

## Abstract

Cardiometabolic multimorbidity (CMM), defined as the simultaneous presence of two or more cardiovascular and metabolic diseases in an individual, including but not limited to type 2 diabetes(T2D), chronic kidney disease(CKD), heart failure(HF), stroke, and obesity, constitutes an expanding global burden that challenges the prevailing single-disease paradigm of contemporary therapeutic interventions. Yet routine care is often guided by single-disease guidelines, yielding treatment plans that are siloed, polypharmacy-heavy, and potentially conflicting. Emerging evidence from large-scale outcome trials (2020–2025) and translational studies demonstrates that pharmacologic agents originally developed for glucose control exert multi-organ protective effects through distinct mechanistic pathways and these agents consistently reduced cardiovascular and renal events beyond glycemic control, with additive benefits when appropriately combined. This review indicates that sodium-glucose cotransporter 2 inhibitors or GLP-1 receptor agonists should be prioritized based on phenotypic characteristics, while Non-steroidal mineralocorticoid receptor antagonist should be considered for use in chronic kidney disease phenotypes. Moreover, the implementation of threshold monitoring protocols is imperative in order to mitigate the risk of hypoglycemia, hypotension, and hyperkalemia. This mechanism-based optimization of therapeutic strategies provides significant guidance for the management of cardiometabolic syndrome and shows promise in improving clinical outcomes for patients suffering from comorbid cardiometabolic diseases. It is recommended that future research concentrate on patient populations with overlapping phenotypes, with a view to refining the decision criteria for treatment de-escalation or discontinuation.

## Graphical Abstract

**Figure:**
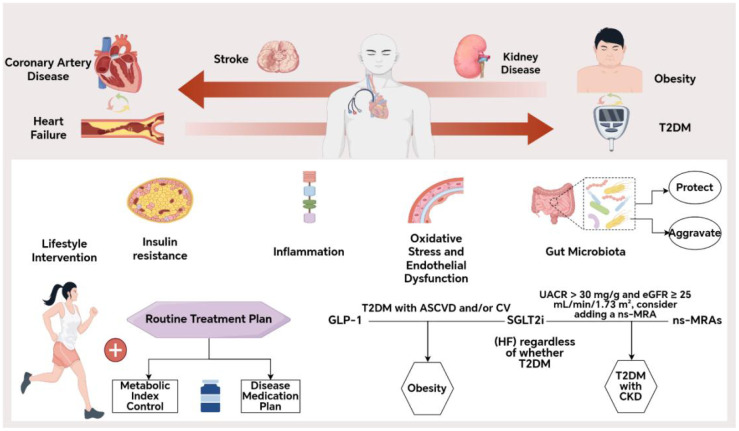

## Highlights

- Mechanism-guided care aligns pathophysiology with phenotype-prioritized pharmacotherapy in CMM.
- SGLT2 inhibitors, GLP-1 receptor agonists, and finerenone deliver multi-organ protection beyond glycemia.
- Combination and sequencing strategies reduce cardiovascular–renal events while curbing polypharmacy.
- Practical de-escalation and safety thresholds (hypoglycemia, hypotension, hyperkalemia) improve net benefit.
- A phenotype-based decision table supports precision prescribing across ASCVD, HF, CKD and obesity.

## Introduction

### The escalating burden of multimorbidity

The global prevalence of cardiometabolic multimorbidity (CMM)—with diabetes at its core—continues to rise and has become a major public-health challenge (1). Projections from the Global Burden of Cardiovascular Disease, 2020–2025, indicate that metabolic risk factors will remain the principal drivers of cardiovascular mortality (2). In a long-term cohort including 1.2 million participants, cardiometabolic multimorbidity was associated with a substantial reduction in life expectancy (3). This clustering of conditions not only markedly increases all-cause mortality, the risk of recurrent cardiovascular events, and hospitalizations, but also imposes a considerable economic burden on health systems. Although a number of clinical practice guidelines for individual cardiovascular or metabolic conditions have been developed both nationally and internationally, most are formulated from a single-disease perspective and lack comprehensive recommendations for multimorbidity management. In clinical practice, physicians often rely on multiple independent guidelines to develop treatment plans for patients with CMM, which may result in polypharmacy, increased risk of drug–drug interactions, decreased patient adherence, and cumulative adverse drug events. Furthermore, cardiovascular and metabolic diseases are pathophysiologically interconnected. Numerous studies have demonstrated that metabolic abnormalities—such as insulin resistance—can significantly influence the onset and progression of cardiovascular diseases (4, 5). However, current clinical strategies rarely adopt an integrated mechanistic approach to managing multimorbidity, making it difficult to simultaneously maximize therapeutic benefit and minimize pharmacologic risk.

Against this backdrop, the present review systematically summarizes recent advances (2020–2025) in the understanding of pathophysiological mechanisms underlying CMM. Special emphasis is placed on the clinical evidence and application of various therapeutic agents, including sodium-glucose cotransporter-2 (SGLT2) inhibitors and glucagon-like peptide-1 receptor agonists (GLP-1 RA), in the management of CMM. This review also seeks comprehensive clinical strategies for the optimization of multimorbidity care. To provide certain guiding significance for the precision medicine of comorbid populations.

## Methods

**Figure 1:**
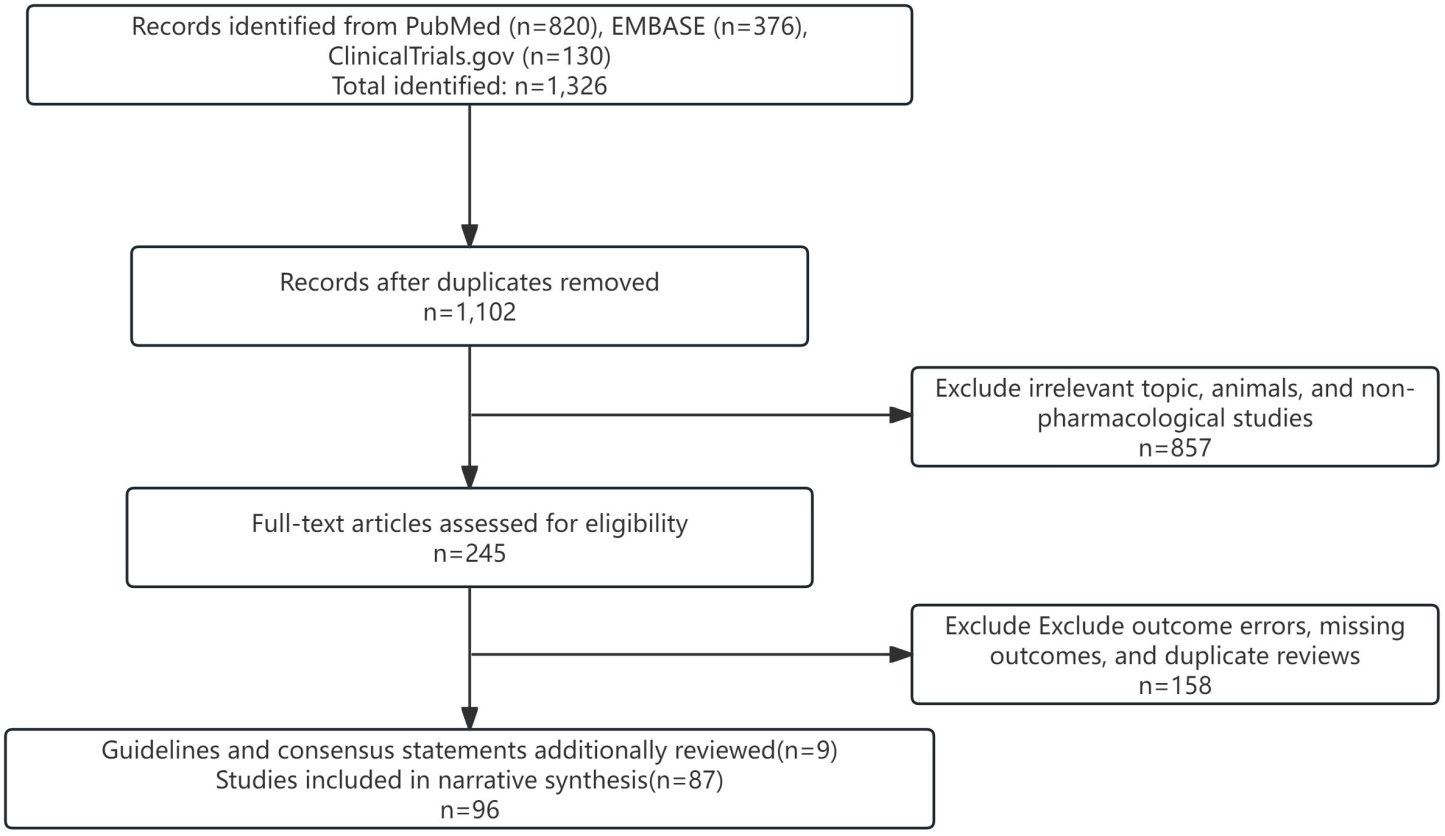
Literature screening process.

To capture high-quality evidence as comprehensively as possible, the authors conducted a structured search of PubMed, Web of Science Core Collection, and Embase for studies published between January 2020 and July 2025. The strategy centered on the core topic and combined Medical Subject Headings (MeSH) with free-text terms, for example:”(‘cardiometabolic multimorbidity’ OR ‘heart failure’ OR ‘coronary artery disease’) AND (‘type 2 diabetes’ OR ‘obesity’ OR ‘dyslipidemia’) AND (‘pathophysiology’ OR ‘mechanism’ OR ‘drug therapy’ OR ‘polypharmacy’ OR ‘deprescribing’).” (**Figure 1**). Complete search syntax are provided in **Supplementary Material S1** for transparency.

Inclusion criteria were published randomized controlled trials (RCTs), meta-analyses, systematic reviews, translational studies, and large observational cohorts; populations with coexisting cardiovascular and metabolic diseases; and a focus on mechanistic insights, pharmacologic interventions, and clinical outcomes. Exclusion criteria were animal studies, case reports, records without an accessible abstract and full text and studies lacking substantive mechanistic or therapeutic findings.

This paper is a narrative review. However, to ensure the quality of evidence in the included studies, three authors independently screened titles and abstracts and critically assessed methodological quality, sample size, consistency of results, and clinical relevance. Disagreements were resolved by the corresponding author. Although a formal PRISMA workflow was not undertaken, the Appendix details the full search strings, deduplication log, and selection notes. Priority was given to pivotal, large RCTs; influential experimental and translational work; high-impact systematic reviews and meta-analyses; and the most recent clinical practice guidelines and consensus statements from relevant professional societies. Evidence levels were assigned using the Oxford Centre for Evidence-Based Medicine (OCEBM) framework (Levels I–V). Certainty ratings were presented in parallel using a simplified GRADE approach (“GRADE-lite”: high/moderate/low), with up-/downgrading based on consistency, precision, indirectness, and risk of bias (**Table 1**).

**Table 1:** Key randomized controlled and cardiovascular outcome trials (2020–2025 window).

No.	Drug/class (year)	Population (key inclusion)	Primary endpoint	Statistic	Effect size	Direction	OCEBM	GRADE -lite
1	Dapagliflozin/SGLT2i (2020) (6)	CKD(with/without T2D); eGFR 25–75; UACR 200–5000	≥50% eGFR reduction/ESKD/renal death	HR (95%CI)	0.56 (0.45–0.68)	↓	I	High
2	Empagliflozin/SGLT2i (2023) (7)	CKD(with/without T2D); broad ranges of eGFR and UACR	kidney disease progression or CV death	HR (95%CI)	0.71 (0.62–0.81)	↓	I	High
3	Empagliflozin/SGLT2i (2020) (8)	HFrEF (± T2D); EF ≤ 40%	CV death or HF hospitalization	HR (95%CI)	0.75 (0.65–0.86)	↓	I	High
4	Empagliflozin/SGLT2i (2021) (9)	HFpEF (± T2D); EF>40%	CV death or HF hospitalization	HR (95%CI)	0.79 (0.69–0.90)	↓	I	High
5	Dapagliflozin /SGLT2i (2022) (10)	HFmrEF/HFpEF (± T2D); EF>40%	HF worsening or CV death	HR (95%CI)	0.82 (0.73–0.92)	↓	I	High
6	Sotagliflozin/SGLT1/2i (2021) (11)	T2D + CKD	CV death/HHF/urgent HF visittotal events	HR (95%CI)	0.74 (0.63–0.88)	↓	I	Moderate
7	Sotagliflozin/SGLT1/2i (2021) (12)	T2D + recently decompensated HF	CV death/HHF/urgent HF visittotal events	HR (95%CI)	0.67 (0.52–0.85)	↓	I	Moderate
8	Finerenone/ns-MRA (2020) (13)	T2D + CKD	Kidney failure/sustained decline in eGFR ≥40%/renal death	HR (95%CI)	0.82 (0.73–0.93)	↓	I	High
9	Finerenone/ns-MRA (2021) (14)	T2D + CKD (earlier stage)	CV composite(CV death/nonfatal MI/nonfatal stroke/HHF)	HR (95%CI)	0.87 (0.76–1.01)	≈	I	Moderate
10	Finerenone/ns-MRA(2022) (15)	T2D + CKD (FIDELIO/FIGARO individual patient data)	CV composite; renal composite	HR (95%CI)	CV 0.86 (0.78–0.95); renal 0.77 (0.67–0.88)	↓	I	High
11	Semaglutide 2.4 mg/GLP-1RA (2023) (16)	ASCVD + overweight/obesity (without T2D)	MACE(CV death/nonfatal MI/nonfatal stroke Moderate)	HR (95%CI)	0.80 (0.72–0.90)	↓	I	High

The time window is 2020-01-01 to 2025-07-31; only original RCTs/CVOTs or prespecified pooled analyses published within this window were included. OCEBM levels per Oxford criteria. GRADE-lite assigned based on simplified rules considering consistency, precision, indirectness, and risk of bias (High/Moderate).

## Mechanistic insights

### The central pathophysiologic role of insulin resistance

Insulin resistance (IR) has long been recognized as a key pathophysiological mechanism underlying both cardiovascular and metabolic diseases (17). The triglyceride-glucose (TyG) index, a reliable surrogate marker for IR, has been shown to be associated with adverse cardiovascular outcomes in patients with diabetes and cardiovascular disease (CVD) (18, 19). Recent studies have not only reinforced this association but also elucidated its downstream effects in greater detail.

Dysregulated lipid-derived metabolic mediators—such as adipokines, excess lipids, and toxic lipid metabolites—released from adipose tissue contribute significantly to the development of IR (20). These adipose-derived factors can impair cardiomyocyte signaling, induce structural remodeling of the heart, and increase the incidence of CVD. They are also closely linked to the pathogenesis of obesity and type 2 diabetes mellitus (T2DM) (21).Excess circulating free fatty acids (FFAs) infiltrate the liver and skeletal muscle, enhancing gluconeogenesis and very low-density lipoprotein (VLDL) synthesis, thereby promoting hepatic and muscular insulin resistance (22). Hepatic IR leads to increased endogenous glucose production and elevated fasting plasmaglucose, which subsequently raises serum triglyceride (TG) levels and lowers high-density lipoprotein cholesterol (HDL-C), heightening thrombogenic risk (23).

In the insulin-resistant state, elevated insulin and aldosterone levels reduce nitric oxide (NO) bioavailability, leading to endothelial dysfunction and pathological arterial stiffening. This process also activates MAPK/ERK-mediated reactive oxygen species (ROS) generation, further increasing cardiovascular risk (17, 24). Additionally, recent evidence indicates that lipid accumulation in pancreaticβ-cells under IR conditions accelerates β-cell apoptosis and senescence, perpetuating a vicious cycle of metabolic deterioration (25, 26).IR is the pathological hub of CMM and links lipotoxicity, endothelial dysfunction and β-cell failure. Therefore, a drug combination that improves insulin resistance and reduces fat/weight serves as the starting point for downstream, multi-organ benefits.

## Activation of the inflammasome pathway

Inflammation plays a central role in the cardiometabolic disease continuum (27). Over the past five years, research has expanded beyond traditional inflammatory markers such as C-reactive protein (CRP), moving toward cellular and molecular mechanisms. The activation of the NOD-like receptor protein 3 (NLRP3) inflammasome has emerged as a pivotal molecular platform linking metabolic stressors—such as cholesterol crystals, hyperglycemia, and FFAs—to the release of proinflammatory cytokines, including IL-1α, IL-1β, and IL-18. These cytokines not only impair glucose metabolism and exacerbate IR (28, 29), but also activate vascular endothelial cells, promote monocyte adhesion, and damage cardiomyocytes, thereby accelerating the progression and instability of atherosclerotic plaques (30).

Immunological studies have further demonstrated that metabolic dysregulation leads to immune cell phenotype shifts and tissue infiltration. Specifically, M1-type macrophage polarization, expansion of pro-inflammatory T-cell subsets (Th1, Th17), and reduction in regulatory T cells (Tregs) have been observed in adipose tissue, liver, vasculature, and myocardium (31). These alterations contribute to local and systemic inflammatory microenvironments, providing strong evidence for the interplay between immunity and metabolic disease progression (32–34). The inhibition of the NLRP3-mediated inflammatory pathway provides a mechanistic basis for the combined use of SGLT2i and ns-MRA in reducing residual inflammation-fibrosis risk.

## Oxidative stress and endothelial dysfunction

Oxidative stress constitutes a shared pathological axis linking inflammation and insulin resistance, further perpetuating their vicious interplay. Under hyperglycemic and hyperlipidemic conditions, mitochondrial production of reactive oxygen species (ROS) increases significantly. At elevated levels, ROS disrupt cellular redox homeostasis and overwhelm endogenous antioxidant defenses (35). Excess ROS directly oxidize DNA, proteins, and lipids, and also activate multiple stress-sensitive kinases and transcription factors such as NF-κB, aggravating both inflammation and IR (36, 37).

Superoxide anions (a form of ROS) rapidly react with endothelial-derived nitric oxide (NO), reducing NO bioavailability and generating highly reactive peroxynitrite radicals (38). This reaction is a hallmark of endothelial dysfunction and is considered a critical initiating event in atherosclerosis and hypertension (39, 40).

## Gut microbiota

In recent years, growing clinical interest has focused on the role of gut microbiota dysbiosis in cardiometabolic diseases. Although causality remains to be fully established, its relevance to the pathogenesis of cardiometabolic multimorbidity is increasingly recognized. The gut microbiota is now regarded as a “quasi-endocrine organ” due to its systemic metabolic influence (41, 42).

Emerging studies have introduced the dietary index of gut microbiota (DI-GM), suggesting that a higher DI-GM score is associated with reduced CMM risk—mediated in part by attenuation of systemic inflammation (43). Fermentation of dietary fiber by beneficial gut bacteria produces short-chain fatty acids (SCFAs), which improve gut barrier integrity and exert systemic effects including anti-inflammatory action, regulation of glucose and lipid metabolism, blood pressure control, and endothelial protection. SCFAs may also have potential roles in mitigating comorbid conditions such as insomnia (44–46).

**Figure 2:**
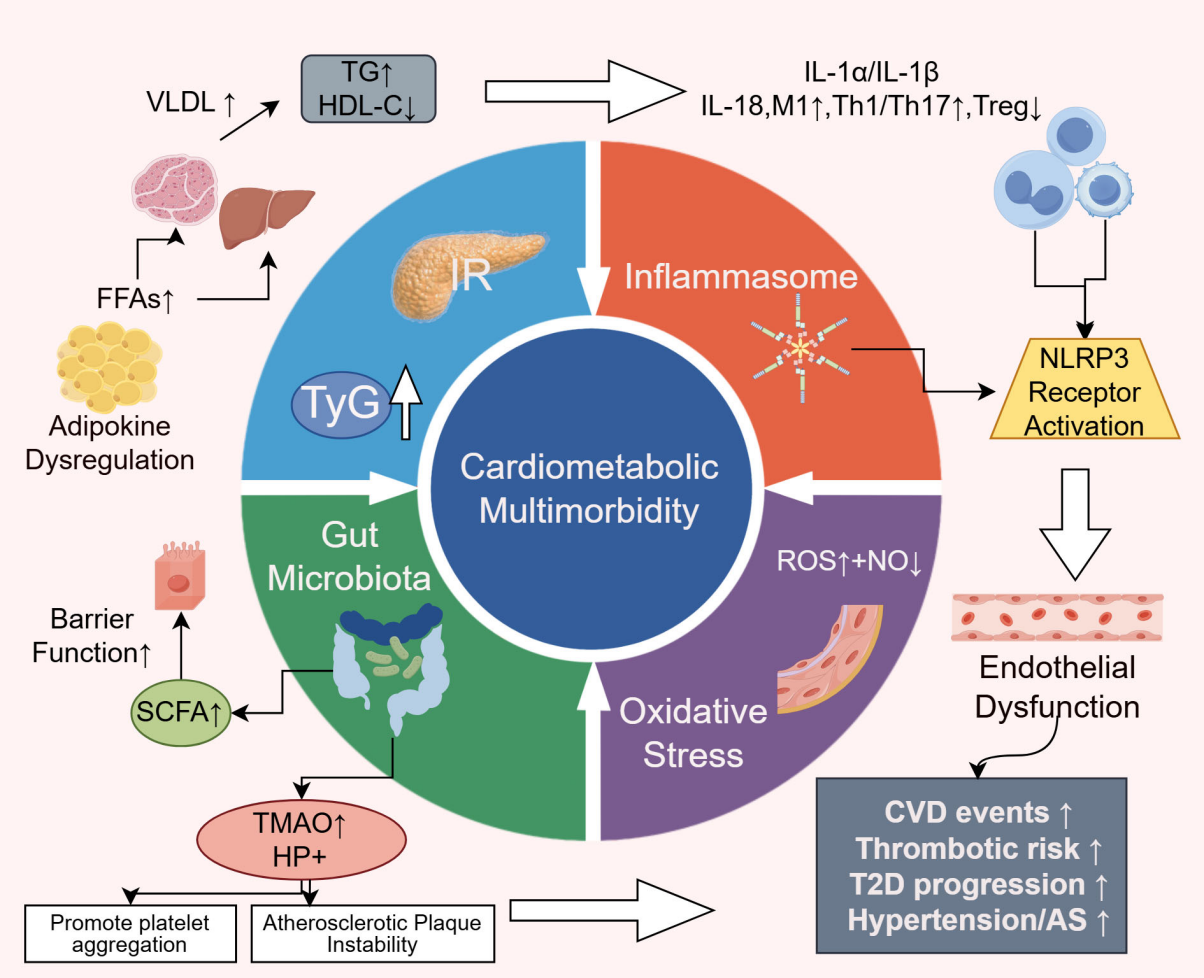
Pathophysiological mechanisms of CMM. IR, Insulin Resistance; TyG: Triglyceride-glucose index; FFAs, Free Fatty Acids; NO, Nitric Oxide; ROS, Reactive Oxygen Species; SCFAs, Short-chain Fatty; TMAO, Acids microbiota-derived trimethylamine N-oxide.

Conversely, elevated levels of microbiota-derived trimethylamine N-oxide (TMAO) and Helicobacter pylori infection have been linked to increased atherosclerotic burden and cardiovascular risk (47, 48). Although the exact mechanism of H. pylori-induced endothelial injury remains unclear, TMAO has been shown to promote platelet aggregation, foam cell formation, and atherosclerotic plaque instability, making it an independent risk factor for CMM (49, 50).The combination of dietary strategies that enhance SCFA production and reduce TMAO load with therapeutic drugs has the potential to positively impact clinical outcomes (**Figure 2**).

## Mechanism-oriented drug intervention programs

### Sodium–glucose cotransporter 2 inhibitors

SGLT2 inhibitors (SGLT2i), such as empagliflozin, dapagliflozin, and canagliflozin, have demonstrated benefits beyond glycemic control, showing pleiotropic effects across multiple organs and systems (51, 52). By inhibiting renal proximal tubule reabsorption of sodium and glucose, these agents induce osmotic diuresis and natriuresis, thereby reducing blood pressure and cardiac preload (53). They also enhance glycemic control and promote weight loss via increased urinary glucose excretion (54).

On the level of energy metabolism, SGLT2i promote substrate shift towards fatty acid and ketone utilization, improving myocardial and renal energy efficiency (55). Additionally, they exhibit anti-inflammatory and anti-fibrotic properties, partly through attenuation of NLRP3 inflammasome activation via Ca^2+^ modulation, thereby directly protecting cardiac and renal tissues (56, 57).

Multiple large-scale clinical trials have shown that SGLT2i significantly reduce the risk of cardiovascular death, heart failure hospitalization, and renal events in patients with T2DM complicated by atherosclerotic cardiovascular disease (ASCVD) or heart failure (58). Notably, these benefits extend to non-diabetic populations, such as patients with heart failure with reduced ejection fraction (HFrEF), in whom substantial clinical improvements have also been observed (59).

### Glucagon-like peptide-1 receptor agonists

GLP-1 receptor agonists, including liraglutide, semaglutide, and dulaglutide, exert multifaceted effects by activating GLP-1 receptors in various tissues beyond the pancreas (9, 60). Their mechanisms of action and clinical benefits include the following:

In glucose and weight control, GLP-1 RA lower blood glucose levels and induce significant, sustained weight loss by centrally suppressing appetite and delaying gastric emptying (61–63). In atherosclerosis prevention, these agents exert direct effects on vascular endothelial cells and immune cells, reducing inflammatory processes within atherosclerotic plaques, improving endothelial function, and lowering LDL-C and blood pressure levels (64, 65).

Cardiovascular outcome trials (CVOTs) have consistently demonstrated that GLP-1 RA reduce the risk of major adverse cardiovascular events (MACE), particularly nonfatal myocardial infarction and stroke (66, 67). However, their efficacy in reducing cardiovascular and renal outcomes among non-diabetic populations remains to be established. Currently, GLP-1 RA are especially recommended for patients with T2DM and established ASCVD or high ASCVD risk, as well as those requiring intensive weight management.

### Nonsteroidal mineralocorticoid receptor antagonists

Finerenone, a next-generation nonsteroidal mineralocorticoid receptor antagonist (ns-MRA), inherits the anti-inflammatory and anti-fibrotic properties of traditional MRAs and offers additional therapeutic benefits. Preclinical and clinical data demonstrate its capacity to reduce fibrosis, lower pulmonary artery pressure, improve diabetic retinopathy, enhance endothelial function, and attenuate oxidative stress, thereby providing comprehensive cardiorenal protection (68, 69).

Finerenone exerts targeted anti-inflammatory and anti-fibrotic effects by selectively blocking mineralocorticoid receptor (MR) overactivation in cardiac, vascular, and renal tissues. This results in marked reductions in biomarkers such as NT-proBNP and UACR, which are closely associated with heart and kidney injury (70, 71). Although mild hyperkalemia may occur during early treatment, it does not negate the overall cardiorenal benefits of the drug. Furthermore, finerenone improves endothelial function, reduces vascular oxidative stress, and restores NO bioavailability, reinforcing its organ-protective profile (72).

Two large-scale trials involving 13,026 patients with chronic kidney disease (CKD) and T2DM confirmed that finerenone, compared to placebo, significantly reduces the risk of CKD progression and cardiovascular events while slowing the decline in estimated glomerular filtration rate (eGFR) (13, 15).

### Other pleiotropic agents

Statins remain the cornerstone of ASCVD prevention and treatment. Beyond their potent lipid-lowering effects, they also exhibit anti-inflammatory, antioxidant, plaque-stabilizing, and vasodilatory properties (73). However, recent studies have indicated that prolonged or high-dose statin use may be associated with an elevated risk of muscle-related adverse effects, hyperglycemia, hemorrhagic stroke, liver disease, and cognitive decline (74, 75). A meta-analysis by Soleimani et al. indicated that moderate-dose statins combined with ezetimibe provide greater LDL-C reduction and fewer adverse events than high-dose statins alone (76), highlighting the need for dose optimization in clinical practice to mitigate adverse effects. Additionally, angiotensin-converting enzyme inhibitors (ACEIs), angiotensin receptor blockers (ARBs), and sacubitril/valsartan not only reduce blood pressure but also confer renoprotective, anti-inflammatory, and endothelial function-improving benefits (77, 78), making them vital components of cardiovascular therapy.

## Optimizing clinical pharmacotherapy: summary and recommendations

### Foundational treatment for patients with cardiometabolic multimorbidity

All patients should initiate and maintain lifestyle interventions, including heart-healthy dietary patterns, regular physical activity, smoking cessation, and alcohol moderation, as the foundation of any pharmacological treatment.

For patients with ASCVD, the initiation of statin therapy at an appropriate dose should be promptly initiated based on baseline LDL-C levels. In instances where LDL-C targets are not met or cases of statin intolerance arise, combination therapy involving ezetimibe or a PCSK9 inhibitor should be contemplated.

For patients with heart failure with reduced ejection fraction (HFrEF), guideline-directed medical therapy (GDMT) should include the “four pillars” of pharmacologic management: β-blockers, ACEIs/ARBs/ARNIs, MRAs, and SGLT2i (**Table 2**).

**Table 2:** Preferred pharmacotherapy based on comorbidity phenotypes.

Phenotype	Clinical features	Preferred pharmacotherapy	Key decision points & notes
A	T2DM with ASCVD and/or high CV risk	GLP-1RA or SGLT2i	If substantial weight reduction is desired or stroke prevention is prioritized, prefer a GLP-1 RA (79, 80). If heart failure (HF), chronic kidney disease (CKD), or reduction of HF hospitalization risk is a concern, prefer an SGLT2i (81). Combination therapy (GLP-1 RA + SGLT2i) may have synergistic/additive effects and further enhance cardio-renal protection (82, 83).
B	T2DM with CKD	SGLT2i (within the labeled eGFR eligibility range)	Combination therapy: if UACR>30 mg/g and eGFR ≥ 25 mL/min/1.73 m², consider adding a nonsteroidal mineralocorticoid receptor antagonist (ns-MRA; finerenone) (84, 85). SGLT2i plus ns-MRA can further reduce residual cardio-renal risk (86).
C	HF,(regardless of whether T2DM is present)	SGLT2i	SGLT2i have been approved for both heart failure with reduced ejection fraction (HFrEF) and heart failure with preserved ejection fraction (HFpEF) (87). They should be combined with other foundational guideline-directed medical therapy (GDMT), e.g., ARNI/ACEI/ARB, β-blocker, and MRA (88).
				D	Obesity + ASCVD without T2D	GLP-1 RA	GLP-1 RA: The initiation of GLP-1 receptor agonist therapy should be based on obesity levels(BMI≥24Kg/m²) and a personal history of ASCVD. The recommendation is that this therapy be initiated promptly rather than being based on blood glucose levels (89).. SGLT2i: may be considered as agents with potential weight-loss benefit. Treatment selection should primarily aim for effective body-weight control (90).

Recommendations assume lifestyle management and individualized care. ASCVD, atherosclerotic cardiovascular disease; ARNI, angiotensin receptor–neprilysin inhibitor; ACEI, angiotensin−converting−enzyme inhibitor; ARB, angiotensin II receptor blocker; CKD, chronic kidney disease; CV, cardiovascular; eGFR, estimated glomerular filtration rate; GDMT, guideline−directed medical therapy; GLP−1 RA, glucagon−like peptide−1 receptor agonist; HF, heart failure; HFrEF/HFpEF, heart failure with reduced/preserved ejection fraction; MRA, mineralocorticoid receptor antagonist; ns−MRA, nonsteroidal MRA; SGLT2i, sodium–glucose cotransporter−2 inhibitor; T2DM, type 2 diabetes mellitus; UACR, urinary albumin−to−creatinine ratio.

### Prioritized drug selection based on comorbidity phenotypes

### Avoiding redundant therapy and ensuring regular medication review

Following the initiation of newer therapies with established multi-organ benefits, such as SGLT2i and GLP-1RA, regular reassessment of the pre-existing treatment regimen is essential. When glycemic and blood pressure control is achieved, clinicians should consider de-escalation strategies, including dose reduction or discontinuation of other antihyperglycemic agents (e.g., insulin) and antihypertensives, to mitigate the risks of hypoglycemia and hypotension.

Establishing a structured medication review schedule is a critical step in optimizing pharmacotherapy. It allows for timely evaluation of drug necessity, minimizes polypharmacy, reduces patient burden, and helps achieve individualized therapeutic goals more safely and efficiently (**Table 3**).

**Table 3:** Indicator-driven simplified table for medication up-titration/down-titration.

Monitoring indicators	Recommendations	Rationale
HbA1c and Blood Pressure = Normal	Evaluating the Reduction of Antidiabetic Drugs and Non-Core Antihypertensive Medications	After adding a new pleiotropic drug, regular monitoring should be conducted; when targets are achieved, the risks of hypoglycemia/hypotension should be reduced by removing redundant drugs.
BG↓ (SGLT2i/GLP-1 RA in use)	Prioritize retention of SGLT2i/GLP-1 RA Down-titrate Ins/SCGN	Orient to organ outcomes, reduce high hypoglycemia-risk meds, Avoid mechanism overlap
SBP↓/orthostatic dizziness (Post new SGLT2i initiation)	Prioritize retention of cornerstone meds with high benefits Decrease dose of hypotensive meds with low benefits or non-cornerstone ones	SGLT2i has proven event reduction in both HFrEF/HFpEF And should be used in combination with GDMT
CKD: UACR >30 mg/g and eGFR ≥25 (SGLT2i in use)	Consider adding finerenone	SGLT2i + ns-MRA can further reduce residual renal and cardiac risks Mechanisms are complementary without overlap
Hyperkalemia during finerenone use	Evaluate finerenone down-titration/discontinuation or intensify monitoring	Finerenone is associated with hyperkalemia risk; safety should be prioritized
Obesity-dominant GLP-1 RA in use (weight/metabolic improvement achieved)	Maintain GLP-1 RA Evaluate and gradually down-titrate Ins/SCGN/hypotensive meds	Target weight control and atherosclerotic risk, Avoid duplication of glucose/lipid-lowering meds and overlapping AEs
ASCVD/high-risk Significant weight loss or stroke prevention required	Prioritize retention/intensification of GLP-1 RA Prefer SGLT2i if HF/CKD is dominant	The two drug classes are complementary: GLP-1 RA (weight loss/anti-atherosclerosis) SGLT2i (heart failure/renal protection)
Polypharmacy	Establish fixed monitoring cycle: Review for potential drug de-escalation after target achievement	Reduce duplicate treatment and multiple AEs, improve adherence

BG, Blood Glucose; Ins, Insulin; SBP, Systolic Blood Pressure; HFrEF, Heart Failure with Reduced Ejection Fraction; HFpEF, Heart Failure with Preserved Ejection Fraction; GDMT, Guideline-Directed Medical Therapy; UACR, Urinary Albumin-to-Creatinine Ratio; eGFR, Estimated Glomerular Filtration Rate; CKD, Chronic Kidney Disease; ns-MRA, Non-steroidal Mineralocorticoid Receptor Antagonist; AEs, Adverse Events; ASCVD, Atherosclerotic Cardiovascular Disease; SCGN, secretagogues.

### Phenotype-based priority treatment across different ages and genders

The management of cardiac metabolic comorbidities must move beyond a one-size-fits-all approach toward precision-based, phenotype-driven treatment strategies that are tailored to specific patient characteristics. Firstly, for elderly patients deemed to be frail, the management plans must be meticulously devised, in view of the complexities arising from polypharmacy and multiple coexisting conditions. This is particularly evident in patients aged ≥75 years with ≥4 chronic diseases, where treatment regimens require a delicate balance between efficacy and safety. It is recommended that streamlined management pathways be established for these patients, centered on the following: 1) Safe medication use: It is imperative to closely monitor and enhance surveillance for the emergence of acute kidney injury, hypovolemia, and hyperkalemia when administering SGLT2 inhibitors in conjunction with NSAIDs or ACEI/ARBs (91). 2) The step-down therapy approach is employed here. It is imperative to establish explicit criteria for the reduction or discontinuation of insulin or sulfonylureas, with the objective of averting hypoglycemic incidents and mitigating the risk of falls and other deleterious outcomes associated with polypharmacy (92).

The existence of gender-based differences in treatment response represents a critical aspect of precision medicine that cannot be overlooked. A mounting body of evidence suggests the presence of gender heterogeneity in the efficacy and tolerability of pharmaceuticals. For instance, subgroup analyses from trials such as DAPA-HF suggest that female patients may experience a greater decline in estimated glomerular filtration rate (eGFR) following SGLT2 inhibitor use compared to males (93). Meanwhile, studies have documented a higher prevalence of gastrointestinal intolerance to GLP-1 receptor agonists in female patients compared to male patients (94). Consequently, when recommending medications based on phenotype, it is imperative to consider gender factors comprehensively. For instance, a more gradual dose titration strategy should be adopted when initiating treatment for female patients, accompanied by enhanced monitoring of renal function and electrolyte levels to optimize treatment adherence and long-term prognosis.

### Equity in mechanism-guided care

In consideration of the national circumstances of differing countries, the “phenotype-first” treatment model that is proposed encounters considerable challenges in resource-constrained settings. In a multitude of low- and middle-income countries, the implementation of novel pharmaceuticals such as SGLT2 inhibitors, GLP-1 receptor agonists, and finerenone may encounter significant challenges due to their substantial financial burden and restricted accessibility (95). This phenomenon, termed the “treatment gap,” has been identified as a contributing factor to global health inequities. In light of this challenge, clinicians and healthcare system decision-makers must not relinquish mechanism-driven treatment principles but rather employ a creative approach in their application to available therapeutic options. The core strategy involves repurposing affordable, widely accessible medications and combining them with enhanced lifestyle interventions. The aim is to mimic or partially replicate the multi-pathway protective effects of novel drugs.

Structured lifestyle interventions constitute the basis of all treatment strategies, a fact that is especially evident in settings with limited resources. Enhanced lifestyle modifications, encompassing a heart-healthy diet, regular physical activity, and smoking cessation, constitute a potent “multi-targeted therapy” capable of simultaneously improving insulin resistance, lowering blood pressure, reducing weight, regulating lipids, and alleviating inflammation (96).When medications are available, priority should be given to novel agents with established organ-protective evidence, such as SGLT2 inhibitors, GLP-1 receptor agonists, and finerenone, to ensure precise targeting of distinct phenotypes. In settings where resources are limited, it is imperative to adhere strictly to maximum tolerated doses of ACEI and ARB in order to reduce proteinuria and slow the progression of renal disease (97, 98). In order to manage cardiovascular risk, it is imperative to combine these with evidence-based statins in order to rigorously control lipids (99).

In instances where finerenone is not available, traditional MRAs remain a vital option based on the same core mechanism. Notwithstanding the necessity for close monitoring of serum potassium and renal function, the addition of spironolactone constitutes a physiologically sound, pragmatic strategy for T2D patients with albuminuria and high cardiovascular risk, following a thorough risk-benefit assessment. It is imperative that the global medical community collaborate to ensure the continuous reduction of barriers to accessing novel therapies. The objective of this initiative is to integrate mechanism-driven advanced treatment concepts with universally accessible healthcare equity, thereby ensuring that patients with CMM—regardless of their geographical location—receive optimal pathophysiology-based care.

## Conclusion

Mechanistic and clinical advances over the past five years have fundamentally transformed the therapeutic paradigm for CMM. Clinical strategies have evolved from a traditional “one-disease, one-drug” approach to an integrated and mechanism-guided model of care. This review underscores the importance of timely and prioritized use of pharmacologic agents with proven multi-organ protective effects—such as SGLT2i, GLP-1RA, and nonsteroidal mineralocorticoid receptor antagonists. These therapies not only target multiple risk factors concurrently but also improve long-term clinical outcomes. Importantly, such integrated approaches facilitate regimen simplification, reduce medication burden and adverse drug events, and emphasize the necessity of precision prescribing. For clinicians, this highlights the critical importance of coordinated and rational pharmacotherapy, ultimately delivering the greatest clinical benefit to patients living with CMM.
