# Supplementary material for: Mechanism-guided pharmacotherapy for cardiometabolic multimorbidity: from pathophysiology to phenotype-prioritized treatment
Source: Front Endocrinol (Lausanne). 2025 Dec 1;16:1724965. doi: 10.3389/fendo.2025.1724965 (PMC12702714; doi:10.3389/fendo.2025.1724965)
Supplement: Supplementary file 1 [file DataSheet1.pdf]

Supplementary Material S1

PubMed Search Strategy

("Diabetes Mellitus, Type 2"[Mesh] OR "type 2 diabetes mellitus"[tiab] OR "type 2 diabetes"[tiab] OR T2DM[tiab]) AND (("Cardiovascular Diseases"[Mesh] OR "atherosclerotic cardiovascular disease"[tiab] OR ASCVD[tiab] OR "heart failure"[Mesh] OR HF[tiab] OR "chronic kidney disease"[tiab] OR CKD[tiab] OR "diabetic kidney disease"[tiab] OR obesity[tiab]) AND ("Sodium-Glucose Transporter 2 Inhibitors"[Mesh] OR "SGLT2 inhibitor*"[tiab] OR empagliflozin[tiab] OR dapagliflozin[tiab] OR canagliflozin[tiab] OR "Glucagon-Like Peptide 1 Receptor Agonists"[Mesh] OR "GLP-1 receptor agonist*"[tiab] OR semaglutide[tiab] OR liraglutide[tiab] OR dulaglutide[tiab] OR "Mineralocorticoid Receptor Antagonists"[Mesh] OR finerenone[tiab]) AND ("Randomized Controlled Trial"[Publication Type] OR "meta-analysis"[Publication Type] OR "cardiovascular outcome trial"[tiab])) AND english[lang] AND ("2020/01/01"[Date - Publication] : "2025/03/31"[Date - Publication]) NOT (animals[MeSH Terms] NOT humans[MeSH Terms]) NOT (editorial[Publication Type] OR letter[Publication Type] OR comment[Publication Type] OR "case reports"[Publication Type])

EMBASE Search Strategy (Ovid)

('diabetes mellitus, type 2'/exp OR 'type 2 diabetes mellitus':ti,ab,kw OR 'type 2 diabetes':ti,ab,kw OR t2dm:ti,ab,kw) AND ('cardiovascular disease'/exp OR ascvd:ti,ab,kw OR 'heart failure'/exp OR hf:ti,ab,kw OR 'chronic kidney disease'/exp OR ckd:ti,ab,kw OR 'diabetic kidney disease':ti,ab,kw OR 'obesity'/exp OR obesity:ti,ab,kw) AND ('sodium glucose transporter 2 inhibitor'/exp OR 'sglt2 inhibitor*':ti,ab,kw OR empagliflozin:ti,ab,kw OR dapagliflozin:ti,ab,kw OR canagliflozin:ti,ab,kw OR 'glucagon like peptide 1 receptor agonist'/exp OR 'glp-1 receptor agonist*':ti,ab,kw OR semaglutide:ti,ab,kw OR liraglutide:ti,ab,kw OR dulaglutide:ti,ab,kw OR exenatide:ti,ab,kw OR 'mineralocorticoid receptor antagonist'/exp OR finerenone:ti,ab,kw) AND ('randomized controlled trial'/exp OR 'meta analysis'/exp OR 'cardiovascular outcome trial':ti,ab,kw) AND [2020-2025]/py AND english/la AND human/lim NOT ('editorial'/it OR 'letter'/it OR 'note'/it OR 'case report'/it) NOT ('animal'/exp NOT 'human'/exp)

ClinicalTrials.gov Advanced Search Fields

Condition or disease: Type 2 Diabetes AND (Atherosclerotic Cardiovascular Disease OR Heart Failure OR Chronic Kidney Disease OR Obesity)

Other terms: (SGLT2 OR empagliflozin OR dapagliflozin OR canagliflozin OR GLP-1 OR semaglutide OR liraglutide OR dulaglutide OR finerenone OR 'nonsteroidal mineralocorticoid receptor antagonist' OR nsMRA OR MACE OR 'cardiovascular death' OR HHF OR 'renal composite' OR ESKD)

Study type: Interventional (Clinical Trial)

Phases: Phase 2–4

Recruitment status: Completed | Active, not recruiting

Study results: All studies

First posted date range: 01/01/2020–07/31/2025

Language: English
